# Clinical practice: Coeliac disease

**DOI:** 10.1007/s00431-012-1714-8

**Published:** 2012-03-16

**Authors:** C. M. Frank Kneepkens, B. Mary E. von Blomberg

**Affiliations:** 1 Department of Paediatrics, VU University Medical Centre, De Boelelaan 1117, 1081 HV Amsterdam, The Netherlands; 2 Department of Pathology, VU University Medical Centre, De Boelelaan 1117, 1081 HV Amsterdam, The Netherlands

**Keywords:** Coeliac disease, Gluten intolerance, HLA-DQ2, HLA-DQ8, Endomysium antibodies, Transglutaminase type 2 antibodies, Small-bowel biopsy, Marsh classification, Gluten-free diet

## Abstract

Coeliac disease (CD) is an immune-mediated systemic condition elicited by gluten and related prolamines in genetically predisposed individuals and characterised by gluten-induced symptoms and signs, specific antibodies, a specific human leukocyte antigen (HLA) type and enteropathy. The risk of coeliac disease is increased in first-degree relatives, certain syndromes including Down syndrome and autoimmune disorders. It is thought to occur in 1 in 100–200 individuals, but still only one in four cases is diagnosed. Small-bowel biopsy is no longer deemed necessary in a subgroup of patients, i.e. when all of the following are present: typical symptoms or signs, high titres of and transglutaminase antibodies, endomysial antibodies, and HLA-type DQ2 or DQ8. In all other cases, small-bowel biopsy remains mandatory for a correct diagnosis. Therapy consists of a strictly gluten-free diet. This should result in complete disappearance of symptoms and of serological markers. Adequate follow-up is considered essential. *Conclusion*: Although small-bowel biopsy may be omitted in a minority of patients, small-bowel biopsy is essential for a correct diagnosis of CD in all other cases. Diagnostic work-up should be completed before treatment with gluten-free diet instituted.

## Introduction

Coeliac disease (CD; gluten intolerance) is an immune-mediated systemic condition elicited by gluten and related prolamines in genetically predisposed individuals. It is characterised by a variable combination of gluten-induced symptoms and signs, specific antibodies, a specific human leukocyte antigen (HLA) type (DQ2 or DQ8) and enteropathy [38]. This new definition, which was very recently coined by a working group of the *European Society for Paediatric Gastroenterology, Hepatology and Nutrition* (ESPGHAN), wanders away from the old one, in which enteropathy (i.e. villous atrophy) had the central role. Yet, intolerance to gluten remains the cornerstone of the definition. Gluten is the collective name for the alcohol-soluble protein fraction of wheat; it is a complex and sticky (hence *glue*) conglomerate of mostly gliadins and glutenins. Prolamins similar to gliadin are also present in other grains, especially in rye (secalins), barley (hordeins), and oats (avenins). In individuals carrying the DQ2 or DQ8 heterodimer, the use of gluten-containing foods may lead to gluten-specific hypersensitivity, resulting in an autoimmune process which induces small-bowel mucosal damage. Complete elimination of gluten from the diet alleviates the immune reaction and provides clinical, serological and histological remission. The diet should be followed for life. The first step in the diagnosis is being acquainted with the disease and recognising the symptoms.

### Beyond CD

Not all persons been found or claiming to experience adverse reaction while consuming gluten or wheat products have CD. This may, however, be mainly applicable to adults. In a very recent consensus paper, an international group of experts distinguished three main groups: autoimmune reactions to gluten, wheat allergy and gluten sensitivity [71]. Autoimmune reactions, all with positive serology, included CD, dermatitis herpetiformis and gluten ataxia. Wheat allergy was primarily represented by bakers’ asthma and exercise-induced wheat allergy, the latter being an IgE-mediated process resulting in acute reactions. Finally, in gluten sensitivity, symptoms and reaction to a gluten-free diet might be indistinguishable from those in CD—although especially neurological symptoms are reported as possible main presenting symptoms [42]—but the characteristic IgA antibodies are missing, small-bowel histology is normal and only about 50 % carry DQ2 or DQ8 [90]. As gluten elimination is the only available method to diagnose gluten sensitivity, much remains uncertain about this condition [71, 90]. For instance, there is much debate about a possible role for gluten sensitivity in children with autism spectrum disorders [22].

## Pathophysiology

### Genetic background

CD is strongly associated with certain human leukocyte antigen class II haplotypes. HLA genes, the human representation of the major histocompatibility complex, are localised on the short arm of chromosome 6 [11]. These genes are involved in the regulation of the immune response. There are two types of HLA-DQ2 molecules, DQ2.5, encoded by the DQ A1*0501/B1*02 allele, with a relative high risk of CD and DQ2.2, encoded by DQ A1*0201/B1*02, with a relatively low risk [84]. DQ2.5 is present in about 95 % of CD patients, with HLA-DQ8 and to a lesser extent DQ2.2 and homozygosity for the HLA-DQ2 β-chain B1*02 accounting for the remaining 5 % [8, 75]. DQ2- and DQ8-positive gluten-specific T lymphocytes recognise certain gluten peptides when presented by antigen-presenting cells. The resulting inflammatory response and tissue damage upregulate the activity of the mucosal enzyme transglutaminase type 2 (TG2; formerly called tissue transglutaminase). DQ2.5-positive antigen-presenting cells cover a larger repertoire of gluten peptides to T cells as compared to DQ2.2 (and DQ8) positive cells; in addition, they bind more stably to gluten peptides [8, 84]. This explains the strong correlation between DQ2.5 positivity and CD.

### Transglutaminase

One of the typical properties of gluten proteins is the excess presence of the amino acids proline and glutamine. Proline is responsible for the compact tertiary structure of gluten. Glutamine residues, carrying an extra amino group, are deamidated by transglutaminase type 2, which is activated as part of the first inflammatory response [80]. DQ2 molecules bind stronger to the resulting glutamic acid-enriched deamidated peptides than to the native peptides, resulting in increased stimulation of T cells; the ensuing enhanced immune response results in an inflammatory reaction in the small-bowel wall. The stages of this process have been carefully established by Marsh [50].

### Marsh classification

**Fig. 1:**
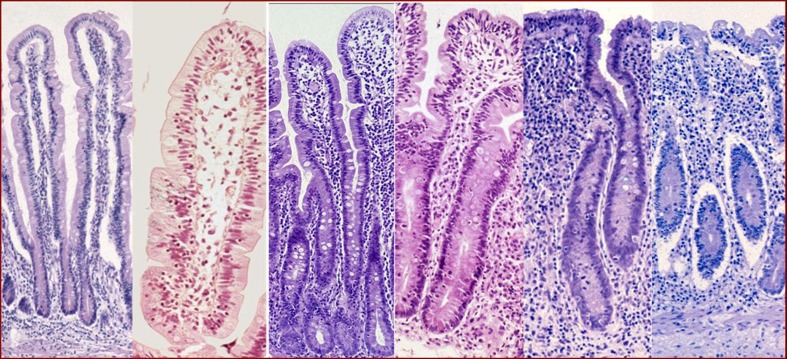
Marsh classification. From *left* to *right*: Marsh 0 = normal; 1 = increase of intraepithelial lymphocytes (IEL); 2 = increased IEL plus crypt hyperplasia; 3a = partial villous atrophy, 3b = subtotal villous atrophy, 3c = total villous atrophy

The influx of T lymphocytes into the epithelium (stage 1) is followed by the destruction of mucosa cells, resulting in increased enterocyte turnover. At first, increased production ensues as witnessed by crypt hyperplasia (stage 2), but when the rate of cell destruction surpasses the rate of cell renewal, also the villi become shorter leading to increasing severity of villous atrophy (partial, stage 3a; subtotal, stage 3b; total, stage 3c; Fig. 1) [50]. Malabsorption of nutrients follows the loss of absorptive surface, which may explain most of the symptoms of CD.

### Other genetic and environmental factors

Although the presence of DQ2 or DQ8, therefore, is more or less a conditio sine qua non, these haplotypes are present in about 40 % of the Western European population, while it is thought that 1 in every 100–200 individuals has CD. So other factors, both genetic and environmental, must be involved in the development of CD. Obviously, a gluten-containing diet is one of these. As far as genetics is concerned, it is estimated that the associated HLA haplotypes represent 36–40 % of the genetic risk of CD [66, 75, 81]. In addition, until now, some 40 genes have been found to be associated with CD, increasing or decreasing the risk of the disease [81, 82]. Interestingly, only one third of the non-HLA genes are exclusively related to CD, two thirds being associated with a host of haematological, metabolic, neurological, oncologic and especially immune-related conditions [81]. Not surprisingly, the most extensive associations were found with type 1 diabetes, rheumatoid arthritis and Crohn’s disease.

It remains mostly unexplained, however, why the age at which CD presents may vary so widely. Being considered mainly a childhood disease until recently, presently the diagnosis is made more often in adults than in children [91]. Possibly the first immune response is elicited by non-specific disturbance of the mucosal integrity, as may be caused by viral gastroenteritis [41, 77].

## Incidence

Increased awareness and improved diagnostic methods have led to a greatly increased incidence of recognised CD. Whereas in the Netherlands, for instance, only 25 years ago, CD was considered a rare disease; the incidence of biopsy-proven CD has been risen from 0.1 to 0.2 per 1,000 life births in the 1970s and early 1980 to 0.54 in 1994 [28] and 1.1 in 2001 [76]. This rise has continued up to the present day: while in the Netherlands in 1994, 105 children were diagnosed with CD; this has risen to 301 in 2010 (resulting in an estimated incidence rate of 1.5 per 1,000) [73]. Yet, this still is the proverbial top of the iceberg, as population-based studies in Western countries, including the Netherlands, have shown that the true prevalence of CD lies closer to 1 per 100 than to 1 per 200 [7, 19, 80]. This implies that for every child diagnosed with CD, four others have not been recognised. Although part of these children may be symptom free, many will have unappreciated symptoms compatible with CD. There are reasons to believe that the increasing incidence of recognised CD is not merely a matter of improved diagnostics, but also reflects a real increase in disease prevalence [47, 70]. As in all autoimmune disorders, girls outnumber boys in CD, with a ratio close of 2:1.

### Effect of HLA type

The relative risk of attracting CD as determined by DQ2 and DQ8 is ‘dose dependent’. The greatest relative risk (8.1) is encountered in individuals homozygous for DQ2.5, the lowest (0.1) in individuals heterozygous for DQ2.2 and DQ8, with a moderate tot substantial (2.1–2.9) risk for DQ2.5 heterozygous individuals [87]. In general, therefore, DQ2 positivity refers to the presence of DQ2.5, whereas DQ2.2-positive individuals are rated ‘DQ2 negative’. Although the latter suggests an extremely low chance of developing CD, our own results suggest that the risk of CD in DQ2.2-positive individuals is comparable to that of individuals carrying DQ8 [57].

### Relatives

As a consequence of the genetic background of CD, the condition is overrepresented in family members. This is particularly true for monozygotic twins, with a relative risk of 17 for the non-index twin to be concordant [31]. About 10 % of CD patients have first-degree relatives with CD. The odds for a family member of having CD, for the greater part depend on the shared HLA haplotype, therefore ranging from <1 % for 40 % of the family members to >20 % for 30 % [9]. In an extensive family study, Megnorni et al. found that CD prevalence was 17.6 % in sisters of CD patients, 10.8 % in brothers and 3.4 % in parents [48]. Siblings with DQ2 or DQ8 had a 20 % chance of having CD (sisters, 29 %; brothers, 15 %), whereas for parents this was 6 %.

### Other groups with increased risk

CD risk is increased in children with certain syndromes, notably Down syndrome [96], Williams syndrome and Turner syndrome [27], as well as in children with autoimmune disorders, especially type 1 diabetes [89]—but also selective IgA deficiency [93], autoimmune thyroid disease [53], autoimmune hepatitis [64] and alopecia areata [60]. In children with type 1 autoimmune hepatitis, the prevalence of CD is reported to average over 20 % [64].

## Signs and symptoms

The clinical spectrum of CD is extremely heterogeneous. The classical picture of a hypotonic, moody toddler with a flat bottom, a huge belly and steatorrhoea may still be seen, but most children present with subtler symptoms or are diagnosed merely as a consequence of screening [67]. The age of diagnosis is extremely variable as well, from infancy, shortly after the introduction of gluten into the diet, to old age. Although symptomatic children generally present with gastrointestinal and malnutrition-related symptoms, this is not always the case. Not only the skin but also the liver, brain, lungs and other organs may be the primary target organ [91]. Additionally, children may present with extraintestinal signs and symptoms that could or could not be related to malabsorption, such as growth failure, anaemia, decreased bone mass [83] and dental enamel defects [94]. A systematic review found evidence for a slightly increased risk in CD children of developing neurological complications, such as headache, peripheral neuropathy and white matter disease [45]. As a consequence, CD presently is considered primarily a systemic disease [38].

Table 1 presents an overview of symptoms and signs associated with CD. Not all of them justify in themselves the inclusion of CD in the differential diagnosis; however, abdominal pain, for instance, seldom, if ever, is the main presenting symptom of CD [26, 29]. There seems to be no correlation between symptomatology and HLA typing [87].

**Table 1:** Symptoms and conditions associated with coeliac disease [38, 44, 59]

Classical presentation
Chronic diarrhoea with abdominal distension, growth retardation, flat buttocks, anorexia and irritability
Atypical presentation
Persistent vomiting, nausea
Recurrent abdominal pain, abdominal distension
Chronic diarrhoea, persistent constipation
Involuntary weight loss
Growth retardation, pubertal delay
Chronic fatigue
Unexplained anaemia (iron, folic acid)
Dermatitis herpetiformis
Other associated symptoms and signs
Osteoporosis, unexplained fractures
Dental enamel hypoplasia
Aphthous stomatitis
Hypertransaminasemia
Polyneuropathy, epilepsy
White matter lesions
Cerebellar ataxia
Associated diseases and syndromes
Type 1 diabetes
Autoimmune thyroiditis
Autoimmune liver disease
Sjögren syndrome
Alopecia areata
IgA deficiency
Down syndrome
Turner syndrome
Williams syndrome

### Silent, latent and potential coeliac disease

Not all children with CD are symptomatic, and not all children with positive antibodies have CD. In the recently published ESPGHAN guideline, the following definitions are proposed for children not fulfilling all criteria for the diagnosis (Table 2) [38]. Children with antibodies, HLA typing and small-bowel biopsy compatible with CD, but without symptoms that would suggest the possibility of CD, are considered to have *silent* CD. This would include relatives of CD patients detected by screening. Children with compatible HLA typing and without actual enteropathy, with or without symptoms and with or without antibodies, but with enteropathy at some other time in their lives, are considered to have *latent* CD. Finally, the presence of antibodies and HLA typing compatible with CD, with or without symptoms, but without enteropathy, is considered *potential* CD, which may or may not turn into actual CD at a later stage [38].

**Table 2:** Active, silent, latent and potential coeliac disease [38]

	DQ2/8	Antibodies	Histology	Symptoms
Active CD	+	+	+	+
Silent CD	+	+	+	−
Latent CD	+	−/+	– (+ in the past)	−/+
Potential CD	+	+	−	−/+

## Diagnosis

The huge variation in manifestations, many of them both non-specific and potentially self-limiting, precludes the use of the response to a gluten-free diet as a diagnostic tool. In young children, for instance, functional diarrhoea, related to a suboptimal feeding pattern, will certainly improve with a balanced diet, whether or not it contains gluten [43]. Moreover, CD is a lifelong condition and therefore deserves a solid diagnosis—which only can be established before treatment is instituted. When, for some reason, a child is put on gluten-free diet before the diagnosis is certain, the only way to prove CD presently is by returning to a gluten-containing diet, thus provoking the signs (and symptoms) of gluten toxicity.

Since the establishment of gluten as the main culprit [85] and villous atrophy as the central abnormality [74], both definition and diagnosis of CD have gone through a number of changes. In 1969, a working group of ESPGAN devised the ‘Interlaken criteria’, requiring three duodenal biopsies: at diagnosis and before and after a period of gluten challenge [54]. These were revised in 1990, when the need of gluten challenge was restricted mainly to children below 2 years of age at the time of diagnosis [92]. The availability of increasingly reliable serologic tests, including transglutaminase type 2 antibodies [12], the notion that even in infants a reliable diagnosis could be made without challenge [95] and changing insight into the basic nature of CD [91] led to another paradigm shift, renouncing the need for biopsy in certain strictly defined situations [38].

### HLA typing

As mentioned before, the great majority of CD patients (95 %) carry the HLA DQ2 heterodimer in *cis* or *trans*, DQ8 being present in most of the remaining patients [75]. In Western populations, however, the prevalence of DQ2 and DQ8 is high (up to 40 % of the general population carry either DQ2 or DQ8, or both). As a result, the positive predictive value of the presence of DQ2 is very low and that of DQ8 even lower, but the negative predictive value of the *absence* of both DQ2 and DQ8 is extremely high, to the point that it virtually excludes CD. The value of HLA typing, therefore, is twofold: on the one hand, it may serve to identify those children from risk groups that might develop CD in the future; on the other hand, it enables the exclusion of CD in part of the patients on gluten-free diet without proper diagnosis. With the new ESPGHAN guidelines, HLA typing has gained a third role, i.e. as one of the criteria for CD in the diagnostic algorithm.

### Serology

Serologic testing is the first step in the diagnosis of CD in symptomatic patients. Up to well into the 1990s, one had to rely on antibodies against gliadin (IgA-AGA, IgG-AGA) and reticulin (IgA-ARA) [5]. These were, however, neither specific nor sensitive enough to be useful in the diagnostic work-up of suspected CD patients. In recent years they have been replaced by tests for antiendomysium (EmA) and anti-TG2 (TG2A). The latter two are extremely specific and sensitive and therefore have positive and negative predictive values that come close to 100 % [72]. The difference between these two antibodies is a relative one, mainly based on different assessment techniques. In fact, transglutaminase type 2 has been identified as the endomysial autoantigen in CD [23]. Interestingly, other transglutaminases may play a role in extraintestinal CD: TG3, expressed in skin cells and found in relation with dermatitis herpetiformis, and TG6, expressed by central nervous system neurons and probably related to gluten ataxia, epilepsy and intracerebral calcifications [9, 42].

Although the level of antibodies is related to the severity of the mucosal lesions, in active CD, as a rule, high titres of IgA-EmA and IgA-TG2A are present. In the new ESPGHAN guidelines, TG2A levels exceeding 10 times the cut-off and confirmed in an independent blood sample by EmA testing are the first requirement for a CD diagnosis without duodenal biopsy in symptomatic children. In IgA-deficient patients, IgG-EmA and IgG-TG2A determination can be used instead, as well as the assessment of IgG antibodies against deamidated gliadin peptides (DGPA). After the institution of a gluten-free diet, all serological markers will disappear, although especially for EmA it may take several years before they disappear completely [36]. In children under 2 years of age, although antigliadin antibodies seem to perform slightly better than EmA and TG2A [49], the new ESPGHAN guidelines discourage the use of these tests and recommend the use of IgG-DGPA instead [38]. The sensitivity of DGPA in this age group seems to be comparable to that of TG2A and EmA [68].

### Small-bowel biopsy

The central role of histological examination in CD has been stressed over and over in the past [25, 35, 54, 62, 69, 92]. The diagnosis of CD was based on the presence of villous atrophy in combination with crypt hyperplasia [54, 92]. A great step forward was made by the introduction of the Marsh criteria [50, 51], which in slightly revised form remain the base of histological interpretation of inflammatory small-bowel lesions (Fig. 1) [52, 62]. The interpretation of small-bowel histology remains to be a challenge, however, and should be performed in an experienced laboratory [4]. Moreover, the diagnosis of CD should never be based on histology alone; the clinical picture, the serology and the effects on both of gluten-free diet should be taken into account as well [17].

The first guidelines required gluten challenge some years after the introduction of gluten-free diet in order to prove the persistence of small-bowel gluten sensitivity [54]. After it was shown that (temporary) villous atrophy due to other causes was found only in infants [32], in the next guideline, the demand for gluten challenge was confined to children younger than 2 years at the age of diagnosis [92]. The subsequent advances in serologic testing as well as more recent data [95], however, prompted most researchers to drop the requirement for gluten challenge in younger children as well.

The next step was challenging the need for small-bowel biopsy. It was shown in several studies that high (>10 times the upper limit of normal) TG2A levels had high positive predictive values for the presence of villous atrophy in children with symptoms suggestive of CD [20, 34, 88]. Similar results were reported in adults [88, 97]. This had led the ESPGHAN working group to develop new algorithms for the diagnosis of CD [38]. It is estimated that these new guidelines lead to the reduction of the need for intestinal biopsies by 20–30 % [38, 97].

In the past it was considered necessary to obtain jejunal biopsies, which required the use of suction capsules [63]. Although already in the 1980s, reports were published confirming the usefulness of duodenal suction biopsies obtained during endoscopy [10], it was only at the end of the last century that forceps biopsies were generally considered adequate [79]. Presently, duodenal biopsies mostly are obtained during endoscopy under general anaesthesia, which is a quick and safe procedure.

### Selection of individuals for diagnostic testing

The symptomatology of CD in children is extremely variable and apart from the now rare ‘classic’ complex of flat-buttocked, large-bellied growth-retarded infants with diarrhoea and bad temper, there is no symptom pattern that strongly suggests the presence of CD. Weight loss, abdominal pain and lassitude have become more prevalent [76], whereas many children express only a single symptom, such as growth retardation and therapy-resistant anaemia (Table 1). In view of the high prevalence of CD, therefore, it is wise to keep a low threshold for serologic testing in children presenting with as yet unexplained symptoms. On the other hand, this will result in a certain percentage of chance findings: children who are proven to have CD but with unrelated presenting symptoms that therefore risk to persist despite the institution of a gluten-free diet. A second group of children to be tested are those with enhanced risk of CD: children with first-degree relatives with CD and children with associated syndromes or other autoimmune disorders, especially diabetes mellitus type 1 (Table 1).

### Algorithms

**Fig. 2:**
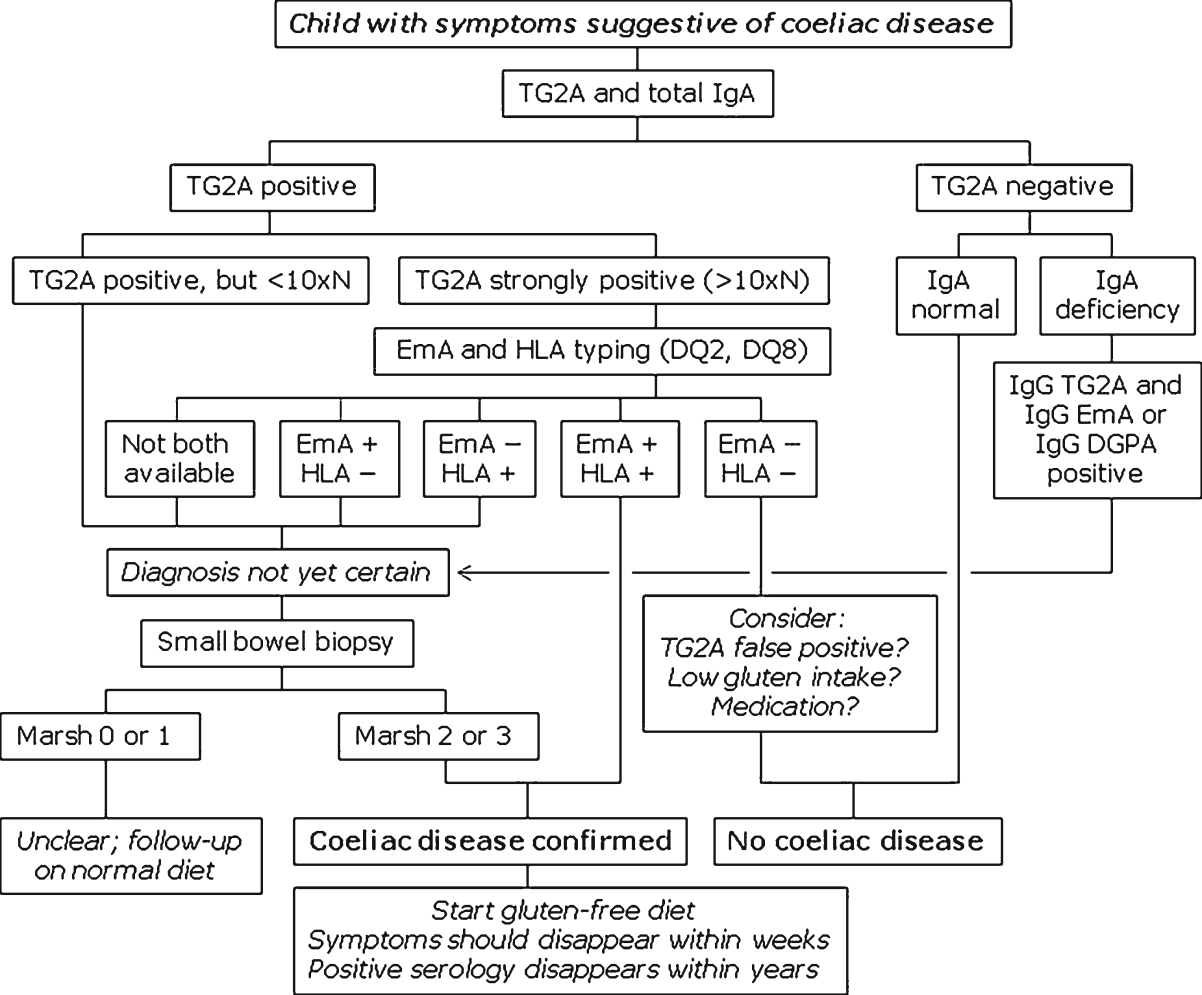
Algorithm for the diagnosis of coeliac disease in symptomatic children. *TG2A* transglutaminase type 2 antibodies, *EmA* endomysium antibodies, *DGPA* deamidated gliadin peptide antibodies, *HLA* human leukocyte antigen, *N* upper limit of normal, *+* present, *−* absent, *Marsh* Marsh classification

**Fig. 3:**
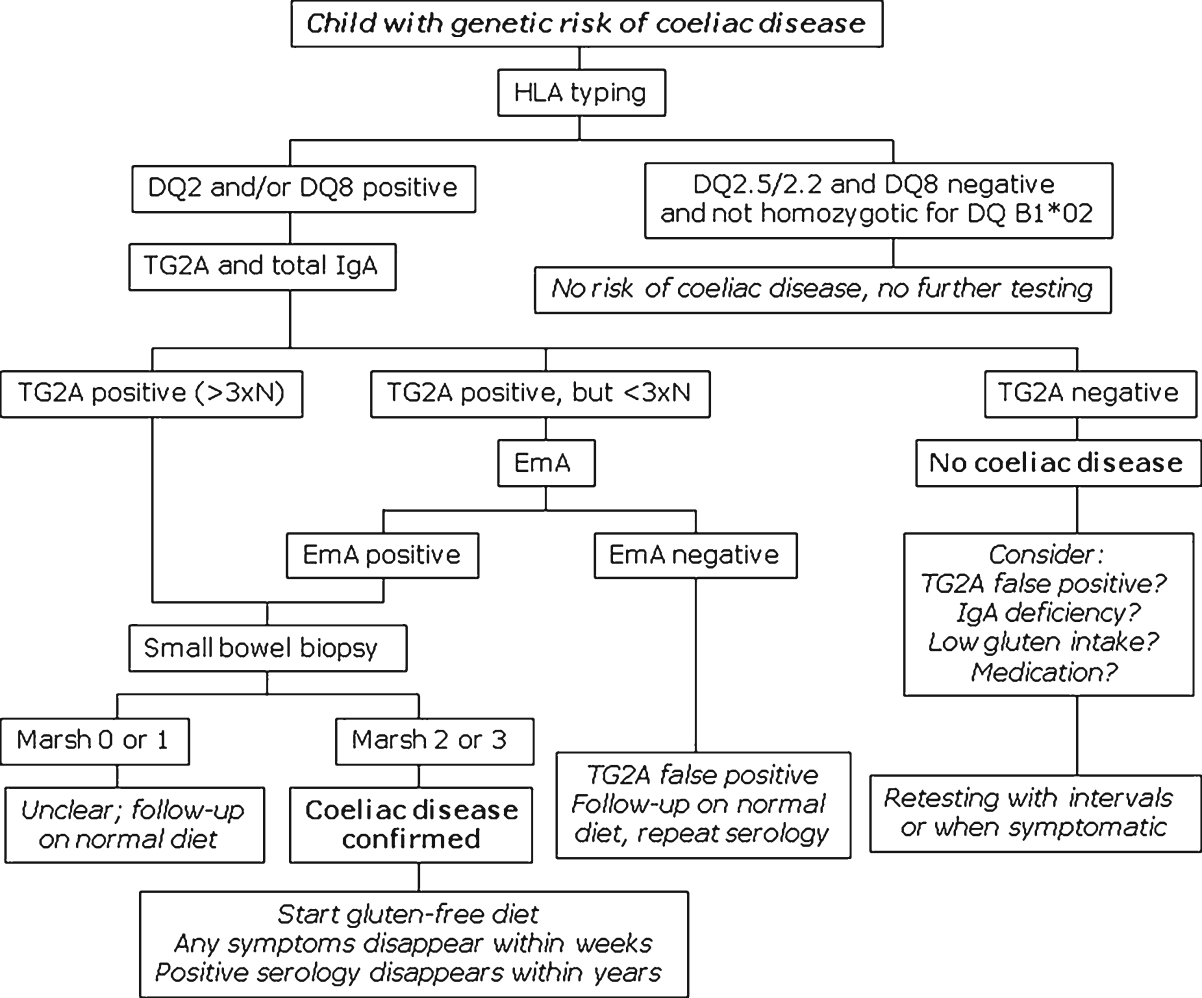
Algorithm for the diagnosis of coeliac disease in children with increased genetic risk and with associated immune disorders. *TG2A* transglutaminase type 2 antibodies, *EmA* endomysium antibodies, *HLA* human leukocyte antigen, *N* upper limit of normal, *Marsh* Marsh classification

Based on current insights into pathophysiology and diagnostic performance, the ESPGHAN working group has developed two algorithms for the diagnostic work-up of CD in symptomatic children and children with increased risk of CD (Figs. 2 and 3) [38]. Important points are the crucial role for HLA typing and the importance of high TG2A levels in combination with positive EmA as a means of eliminating the need of biopsy in a subset of children. Different from the new ESPGHAN guidelines, however, we prefer to take all the necessary blood samples during the first visit, which is more acceptable to the child and the parents. Depending on the results of TG2A testing, we proceed with other tests following the algorithm. It should be noted that in children with increased risk but without typical symptoms, duodenal biopsies are always necessary (Fig. 2).

### Gluten challenge

With the new insights into CD, there is no need to perform gluten challenge unless there is serious doubt about the diagnosis of CD. Gluten challenge still should be performed in children with villous atrophy consistent with CD, but without positive serology, as there are other conditions leading to villous atrophy [38]. Gluten challenge is also inevitable in children who are placed on a gluten-free diet without proper diagnostic work-up. A third group soliciting for gluten challenge are the (older) children who deny the diagnosis because they experience no adverse effects when consuming gluten-containing snacks. With proper understanding, they may accept their diet when they are confronted with the detrimental results of gluten consumption. Challenge should not be performed before the age of 5 years and during puberty [38].

## Treatment

At present, a strictly gluten-free diet is the only available effective treatment for CD. It is, however, virtually impossible to maintain a diet that is completely devoid of gluten, as food is commonly contaminated with gluten. In many commercially available products, ranging from sauces and sausages to soups and chips, gluten is used as a protein filler. In addition, the omnipresence of wheat and wheat starch in production lines may lead to cross-contamination of cereals and other originally gluten-free products [13]. It is, therefore, extremely difficult to achieve a diet completely devoid of gluten. From a practical point of view, products may be called gluten free when they are tolerated by the great majority of CD patients—tolerated not only in terms of symptoms but also of small-bowel histology and quality of life. Unfortunately, there seems to be a grey zone of gluten tolerance between CD patients. While some patients may tolerate about 35 mg gluten per day [16], others seem to be intolerant to smaller amounts [1]. For practical reasons, these daily amounts have to be translated into food product concentrations.

### Definition of ‘gluten free’

With increasing sensitivity of determination techniques for traces of gluten in foods, the threshold for what is allowed to be called gluten free is changing. The *Codex Alimentarius* standard on gluten-free products has been revised in 2008, allowing gluten concentrations in consumer foods labelled gluten free up to 20 mg/kg (20 ppm), analogous to 10 mg prolamin per kg—a reduction to 10 % of the formerly accepted level [15]. ‘Gluten-free’ wheat starch has been shown safe for use to the majority of CD patients [65]. With this threshold, complete adherence to a gluten-free diet will result in gluten intakes amply below 10 mg per day. A second limit—100 ppm—is used to designate foods with very low gluten content. These should, however, not appear on the menu of CD patients.

### Oats

Traditionally, wheat, rye, barley and oats are considered the grains that should be avoided by CD patients. Probably, however, oats used in early studies was heavily contaminated with other grains [24]. Recently, oats has been at least partially acquitted from being harmful to CD patients [3]. In vivo studies confirm that contamination-free oats is not harmful to at least the majority of CD patients [33, 37]. Specially grown and processed oats could, therefore, be included in the gluten-free diet of CD patients, provided that they be followed closely [24].

### Future therapeutic options

Although gluten avoidance presently is the only viable option for the treatment of CD, the increasing knowledge of the pathophysiology of CD has created the perspective of different therapeutic approaches, including decreasing gluten exposure, diminishing intestinal permeability and modulation of the immune response [44]. Especially the use of AN-PEP, a prolyl endoprotease derived from the fungus *Aspergillus niger*, which is capable of instantaneous degradation of gluten within the acid environment of the stomach, seems promising [55]. None of these novel strategies, however, is supposed to completely abrogate the need of following a gluten-free diet [18].

## Follow-up

**Fig. 4:**
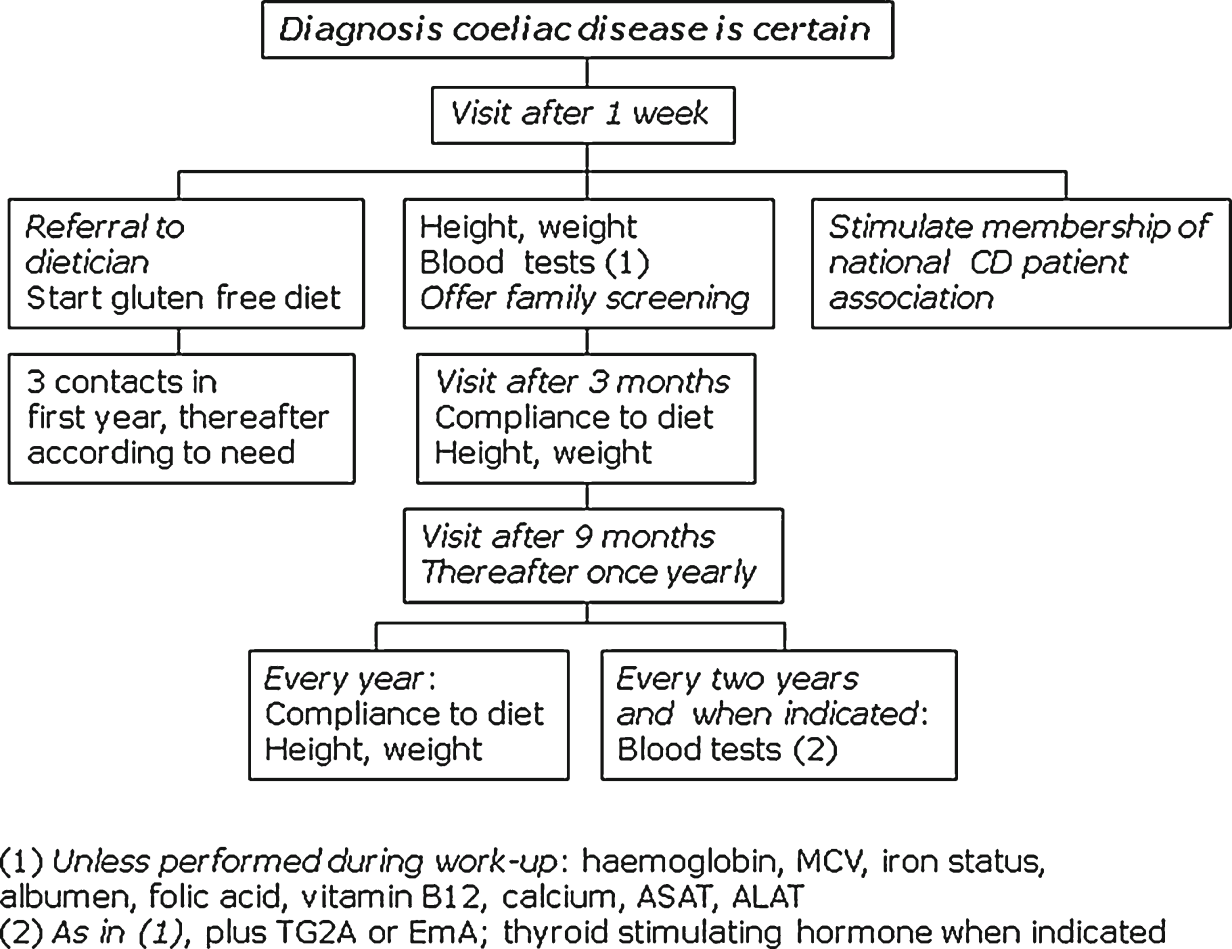
Follow-up of coeliac disease

There is general consensus that children following a gluten-free diet should be followed up on a regular basis. Both the paediatrician and the dietician should be involved. The dietician is familiar with all practical aspects of the diet, including facets as how to read labels of food items, how to keep a corner of the kitchen gluten free and how to prevent contamination when cooking for the whole family. The paediatrician follows growth and well-being of the child, identifies secondary problems and performs blood tests, including follow-up on serology and thyroid function tests when indicated (Fig. 4). The gluten-free diet is relatively low in fibre, and many children experience some defecation problems with constipation or abdominal pain in the first months after starting the diet. Usually this is easily controlled by prescribing macrogol for a few months.

One critical point that should be addressed regularly, especially during puberty and when children exchange primary school for high school, is adherence to the diet. The more the parents are confident that the gluten-free diet is essential for their child’s well-being, the better the diet is accepted by the child. But during puberty many children may start to experiment with gluten-containing snacks, in an attempt not to draw attention to their peculiar diet. Although this may be inevitable, it may help to openly discuss the challenges the child has to cope with. This may also be a reason to propose a formal gluten challenge, in order to convince the patient of the necessity of the diet.

## Prognosis

In children, an adequate diet will invariably result in complete disappearance of symptoms, including full recovery of predicted height growth [21], and serological and histological normalisation. While clinical symptoms usually dissolve within a few weeks (although catch-up growth may only be completed after several years), it may take some years for histology and especially serology to return to normal. Refractory CD, with persistence of villous atrophy, which is seen in 2–5 % of CD patients diagnosed in adulthood [78], is extremely rare in children [56]. Type II refractory CD presents with aberrant intraepithelial lymphocytes and is associated with enteropathy-associated T-cell lymphoma [78]. Several reports mention increased mortality in CD patients; it is thought that the risk of mortality decreases over time with strict adherence to a gluten-free diet [6, 30].

## Prevention

The earliest suggestion that infant feeding practices might have a role in the epidemiology of CD is to be found in letters to the *Lancet* in 1980 [46]. The authors reported a decline in (classical) CD cases after the recommendation of later cereal introduction and the encouragement of breast feeding. Although subsequently it was suggested to be mere postponement of symptoms, the analysis of an epidemic of CD in children under 2 years of age in the Swedish birth cohorts of 1984–1996 confirmed the impact of infant feeding practices on CD incidence [39, 40]. In the 1993 Swedish birth cohort, investigated at the age of 12 years, a total incidence of CD was found of 3 %, three times higher than expected, confirming the epidemic nature of the rise in incidence [58]. A meta-analysis of these and other studies confirmed that both length of breastfeeding and breastfeeding at the time of introduction of gluten offer protection against CD [2]. In addition, the timing of gluten introduction seems to play a role as well, with the smallest risk of CD development obtained with the gradual introduction of gluten between 4 and 6 months of age [61]. These results could be explained by the presence of non-degraded gliadin as well as gliadin–antigliadin IgA complexes in breast milk [14], which could help infants at risk to develop tolerance to gluten [86].

## Conclusion

Better insight into the pathophysiology and better diagnostic tools have led to changes in the diagnostic work-up of children suspected of CD. In around one quarter of symptomatic children, under strictly defined conditions, small-bowel biopsy may be omitted. Given the persistence of gluten sensitivity throughout life and the burden of (unnecessary) dietary treatment, however, utmost care should be exerted when diagnosing this condition and proper follow-up is essential.
